# Effects of Nanofillers Based on Cetyltrimethylammonium-Modified Clays in a Polypropylene Nanocomposite

**DOI:** 10.3390/polym14194110

**Published:** 2022-09-30

**Authors:** Hyeon-Ju Ryu, Nguyen Thu Hang, Sanoj Rejinold. N, Byeongmoon Jeong, Goeun Choi, Jin-Ho Choy

**Affiliations:** 1 Department of Chemistry and Nanoscience, Ewha Womans University, Seoul 03760, Korea; 2 Intelligent Nanohybrid Materials Laboratory (INML), Institute of Tissue Regeneration Engineering (ITREN), Dankook University, Cheonan 31116, Korea; 3 College of Science and Technology, Dankook University, Cheonan 31116, Korea; 4 Department of Nanobiomedical Science and BK21 PLUS NBM Global Research Center for Regenerative Medicine, Dankook University, Cheonan 31116, Korea; 5 Division of Natural Sciences, The National Academy of Sciences, Seoul 06579, Korea; 6 Department of Pre-Medical Course, College of Medicine, Dankook University, Cheonan 31116, Korea; 7 International Research Frontier Initiative (IRFI), Institute of Innovative Research, Tokyo Institute of Technology, Yokohama 226-8503, Japan

**Keywords:** polypropylene, organo-clay, nanocomposites, thermal stability, mechanical strength

## Abstract

Nanocomposites of hydrophobic organo-clay/polypropylene (organo-clay/PP) were efficiently developed through a solution-blending technique. For this, we utilized various smectite clays as host agents; namely, Na-montmorillonite (Mt, ~1000 nm), Na-fluorine mica (Mica, ~1500 nm), and Na-hectorite (Ht, ~60 nm) with varied sizes, layer charges, and aspect ratios. Such clays were functionalized with cetyltrimethylammonium (CTA) bromide via an intercalation technique to obtain hydrophobic organic clays. The as-made clay particles were further mixed with a PP/xylene solution; the latter was removed to obtain the final product of the CTA-clay/PP nanocomposite. An X-ray diffraction (XRD) analysis confirmed that there were no characteristic *(001)* diffraction peaks for CTA-Mica in the PP nanocomposites containing CTA-Mica, assuring the fact that the Mica layers could be completely exfoliated and thereby homogenously composited within the PP. On the other hand, the CTA-Mt and CTA-Ht incorporated composites had broader *(001)* peaks, which might have been due to the partial exfoliation of CTA-Mt and CTA-Ht in the composites. Among the three CTA-clay/PP nanocomposites, the CTA-Mica nanohybrid showed an enhanced thermal stability by ~42 °C compared to the intact host polymer matrix. We also noted that when the CTA-Mica content was ~9 mass % in the nanocomposites, the Young’s modulus was drastically maximized to 69%. Our preliminary results therefore validated that out of the three tested clay-PP nanocomposites, the CTA-Mica nanofiller served as the best one to improve both the thermal and mechanical properties of the PP nanocomposites.

## 1. Introduction

Polypropylene (PP) has been widely applied as a smart polymer in various applications, including thermoplastics, with an improved mechanical stability. They have a low density, are inexpensive, and have a high melting temperature with an ideal mechanical strength for constructing hybrid systems for many applications due to its easy processability [1,2,3,4]. Though PP has many such benefits, its applications, especially for thermostable materials, are restricted by its poor stiffness to some extent. In addition, its alkane molecular structures are comparatively labile for easy burning, thereby restricting its application as a flame-retardant material. Such limitations can be surpassed to some extent by incorporating a suitable nanofiller effectively. Though there are different nanofillers, the clay-based type has a specific advantage in improving the flame retardancy once it is composited with the base polymer materials [5,6]. The overall improvements in the thermomechanical characteristics of such clay/polymer nanohybrids can be ascribed to the uniform distribution of nanofillers across the polymer chains due to their higher surface characteristics and volume ratio, by which they can engage in better interaction with the polymer segments [7,8,9,10].

PP-based composites have been widely explored in various technical fields due to their outstanding mechano-chemical characteristics along with their processability. Nevertheless, due to their poor surface properties, active reaction sites, and high photo/thermal oxidizability, PP hybrids are limited to certain applications. To overcome this, nanofilled PP has been developed using various methods such as plasma treatment, chemical conjugation, and nanoassisted synthesis via coatings or fillings across the PP matrix to enhance the thermo-mechanical properties. There have been reviews on nanofilled PPs for such improved applications [11].

It has been well reported that the incorporation of inorganic-based nanofillers such as Mt/mica/Ht, layered double hydroxide (LDH) and carbon nanotubes could enhance the overall stability of polymer composites [12,13,14,15,16]. In particular, the layered silicates such as Mt/mica/Ht have been utilized as potential additives to develop polymer-based hybrids due to their surface characteristics (a large volume ratio and surface area) in layered smectite clays [16,17,18]. Many attempts have already been made to find a correlation between various factors such as organic clay dispersion, layer charge, particle size, clay aspect ratio, and surfactant in the polymer matrix, but no clear evidence has been provided yet. Therefore, we attempted to compare and analyze Mt (0.40 e^−^/unit cell, ~1000 nm, 250), mica (0.65 e^−^/unit cell, ~1500 nm, 1000) and Ht (0.20 e^−^/unit cell, ~60 nm, 30) with three different layer charges, particle sizes, and aspect ratios of clay among layered silicates.

The layered arrangements (with a thickness of ~1 nm and a high aspect ratio of ~30 to 1500 nm) along with a properly exfoliated nature entails a platelet-like clay particles with a high stiffness and strength that can be homogeneously blended along the polymer chains [16,18,19]. Additionally, the hydrophilic smectite clays can easily be combined with hydrophilic polymers. However, to blend these with engineered polymers, the hydrophilic smectite clays initially should be changed into organophilic ones [14]. Such a modification is possible through a cation exchange reaction of smectite clays with cetyltrimethylammonium (CTA) and similar organic cations [20,21,22]. However, while the effects of nanofillers on PP nanocomposites in improving physico-chemical properties have been demonstrated in detail, little is known about their correlation with the thermomechanical properties of the nanocomposites. In particular, how the physical characteristics (charged layers, nano size of fillers, aspect ratios, loading quantity of nanofillers, and the degree of distribution across the host polymer) could alter the overall stability of organo-modified nanoclays in PP chains are addressed in this manuscript.

It should also be noted that a polymer nanocomposite’s characteristics rely on the processing method through homogeneous mixing of nanofillers across the polymer matrix [12,23]. The major preparation techniques for organo-clay nanocomposites are in situ polymerization, melt blending, and solution blending [4,16,24,25,26]. Among these, solution blending has been effective for uniformly mixing organo-clays in PP chains; therefore, the present study utilized the solution-blending method when designing the clay/PP nanocomposites.

Therefore, three representative smectite-type clays (Mt, mica, and Ht) were selected for the synthesis of the CTA-modified clays. The main objective of the present study was to determine how such organo-clay-based nanofillers could improve the thermal and mechanical properties of a PP composite according to their dispersibility, particle size, and exfoliation rate within the PP matrix.

## 2. Materials and Methods

### 2.1. Materials

The smectite clays used were: mica (Na-fluorine mica: SOMASIF ME-100) from CO-OP Chemicals, Tokyo, Japan, Mt (Na-montmorillonite: Kunipia-F) from Kunimine Industries Co., Ltd., Tokyo, Japan, and Ht (Na-hectorite) from Southern Clay Products, Inc., Gonzales, TX, USA; these were used as obtained (refer to Table S1 for their compositions). The CTA bromide salt C_19_H_42_BrN (M = 364 g/mol) (Sigma, Ltd.) and polypropylene (PP, PP-H1500) were purchased from Lotte Chemical, Co., Ltd., Seoul, South Korea. The xylene (>80%) and ethanol (>99.5%) were purchased from Daejung Co., Ltd., Seoul, South Korea.

### 2.2. Synthesis of Organo-Clays

Our previous method was utilized for intercalating modified clays with CTA molecules [15]. Briefly, 2 g of clay was initially mixed with deionized water (DIW) (100 mL); thereafter, CTA identical to the cation exchange capacity (1.0 CEC) of the clay was gently mixed in. The as-made solutions were kept for 12 h while stirring at 60 °C. Finally, the products were obtained by centrifuging the solutions and then a thorough washing with DIW was done until the bromide and unreacted salt had fully drained out. The final steps involved the vacuum drying of the slurry at 100 °C to obtain the powdered organo-clays, which were called CTA-Mt, CTA-Mica, and CTA-Ht, respectively.

### 2.3. Synthesis of Nanocomposites

As mentioned previously, a solution-blending technique was utilized here. Firstly, 5 g of PP was dispersed in xylene (30 mL) at ~120 °C for 4 h. Various organo-modified clays (1~12 mass %; 0.05 g~0.60 g) were also dissolved in toluene (30 mL) separately. Each of the organo-clay/toluene and PP/xylene reactants were blended well at 120 °C under reflux conditions for 24 h with vigorous stirring; thereafter, the solvents were evaporated using vacuum drying at 80 °C. The formed nanohybrids were labeled based on the type of nanofillers they contained; accordingly, they were marked as follows: CTA-Mt/PP, CTA-Mica/PP, and CTA-Ht/PP with (x) mass % (x = 1, 3, 6, 9, or 12 mass %).

### 2.4. Characterizations

The XRD analysis for all the samples was conducted using a Rigaku X-ray diffractometer ((Cu Kα radiation (λ = 1.5418 Å) at 40 kV and 30 mA)) with a scan rate of 2°/min. The CTA-unmodified clays and CTA clays were measured using XRD in a powder state; nanocomposites were measured using a thick sample. Fourier-transform infrared spectroscopy (FT-IR) spectra of the organo-clays were collected in the range of 400–4000 cm^−1^ using the KBr disk method. Transmission electron microscopy (TEM) images were obtained using a JEM-2100F (JEOL, Tokyo, Japan). Organo-modified clay/PP nanocomposite samples at 6 wt % for TEM analysis were obtained using a cryo-ultramicrotome (RMC CRX) in which the sectioning was conducted at −120 °C with a sample thickness between 60 nm to 80 nm and a cutting speed of 0.3 mm min^−1^.

The thermogravimetric analysis (TGA) of CTA clays/PP nanocomposites was conducted using a TA machine in air (flow rate ~200 mL/min, temperature range of 50–1000 °C, and heating rate of ~5 °C/min). Dynamic light scattering (DLS) (Nano ZS, Malvern, UK) was used to determine the particle size distribution. Tensile tests of the nanocomposites were carried out according to the ASTM D 638-03 standard using a universal testing machine (Zwick Co., Ulm, Germany) at a crosshead speed of 80 mm/min and a temperature of 190 ± 2 °C. Five measurements were conducted for each sample and the results were reported as the averaged values for: (a) barrel temperature (190 ± 2 °C), (b) injection velocity (45 mm/s), (c) screw rotational speed (200 rpm), and (d) holding pressure (65 bar); while the injection pressure and cooling time were set at 75 bar and 50 s, respectively. For mechanical assessment of samples (dumb-bell shaped), a thickness of 3.1 mm and a width of 3.2 mm were used.

## 3. Results

### 3.1. CTA-Modified Clay

#### 3.1.1. XRD Characterization

**Figure 1:**
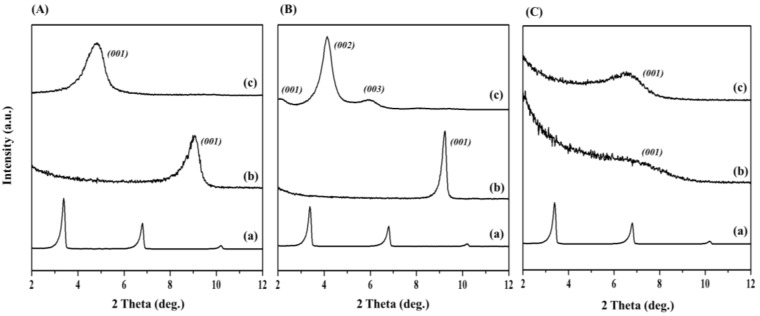
XRD patterns for (**A**) (**a**) CTA, (**b**) unmodified Mt, and (**c**) CTA-Mt; (**B**) (**a**) CTA, (**b**) unmodified mica, and (**c**) CTA-Mica; and (**C**) (**a**) CTA, (**b**) unmodified Ht, and (**c**) CTA-Ht.

The molecular structural information for CTA and PP (Figure S1) and their interaction within the interlayer nanospace of the smectite clays with varied layer charges are also demonstrated in Figure S2. As shown in Figure 1A(b), the intrinsic *(001)* peak for unmodified Mt, which represented the basal spacing of 9.9 Å, completely vanished. Figure 1A(c) shows a new peak at about 5° with d = 20.2 Å. Additionally, we observed that the CTA molecules were well intercalated into the interlayer nanospaces of Mt via a pseudo-triple-layer structure (Figure S2a) [27,28]. In the case of the CTA-modified Mica, the interstratification between the parallel bilayer (18.2 Å) and the pseudo-triple layer (22.7 Å) arrangements from the organic moieties (Figure 1B(c)) and (Figure S2b) could lead to a poor *(001)* peak at the 2θ value of 2.16 (d*_001_* = 40.9 Å). Such a sequential layered arrangement of bilayers across the C-axis showed a heterogenic layer charge in the mica.

It should also be noted that the mica we tested in our experiments contained layered silicates with a sequential arrangement of two charged layers (one with a higher charge and another with a low charge), forming a heterostructure [29]. On the other hand, basal spacing at 12.3 Å (Figure 1C(b)) for the unmodified clay was also expanded to 15.1 Å (Figure 1C(c)) after intercalating with CTA; the organic intercalant was found to have parallel monolayer structural arrangement within the interlayer nanospaces of the host lattice that was ascribed to a lower charge density and distribution (Figure 1(c) and Figure S2c) [30,31,32,33,34].

#### 3.1.2. Fourier-Transform Infrared Spectroscopy Analysis

**Figure 2:**
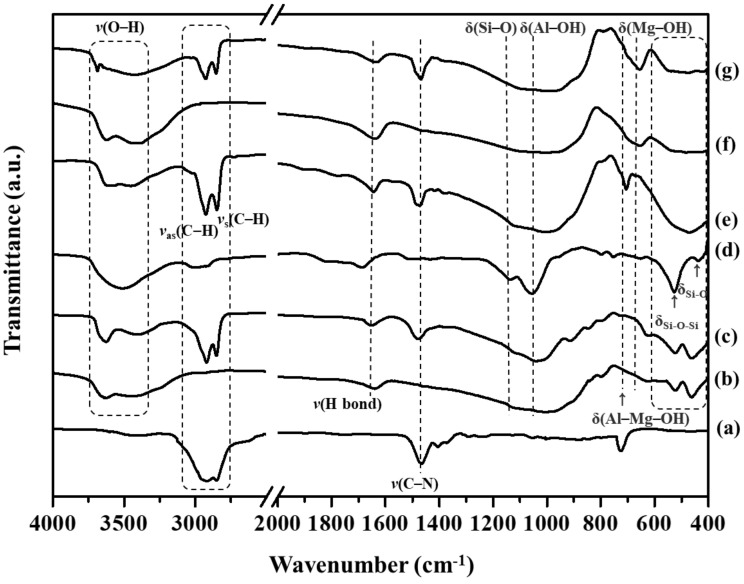
FT-IR spectra of (**a**) CTA, (**b**) unmodified Mt, (**c**) CTA-Mt, (**d**) unmodified mica, (**e**) CTA-Mica, (**f**) unmodified Ht, and (**g**) CTA-Ht.

The assignments of the FT-IR bands for the CTA, unmodified clays, and CTA clays are listed in Table 1. As can be seen in Figure 2a, the CTA’s’ broad bands at 2930 cm^−1^~2840 cm^−1^ were attributed to the antisymmetric C-H stretching vibrations from alkyl groups [20,35,36]. The sharp band at 1470 cm^−1^ represented C-N stretching. On the other hand, the O-H stretching for various tested samples could be observed at 4000 and 3500 cm^−1^ [20]. All three of the smectite clays had their intrinsic bands at 3500~3630 cm^−1^ (Al-OH-Al), 3421~3000 cm^−1^ (H_2_O), 1640~1633 (H-bonds), and 1137~1091 cm^−1^, respectively, which were assigned to the stretching vibration; and at 1055~956 cm^−1^ (Al-OH), 797~787 cm^−1^ (Al-Mg-OH), 655~625 cm^−1^ (Mg-OH), 542~523 cm^−1^ (Si-O-Al), and 459~433 cm^−1^ (Si-O, Si-O-Si), which were assigned to the bending vibration [20,37]. After modification, the CTA clays revealed new bands at ν(Al-OH-Al), ν(H -bonds), ν(Si–O), δ(Al-OH), δ(Al-Mg-OH), δ(Mg-OH), δ(Si-O-Al) and δ(Si-O, Si-O-Si), along with intrinsic IR bands corresponding to the intact clays. The two major new bands from the CTA clays could have been due to C-H and C-N stretching vibrations, which would confirm the proper organo-addition of CTA in the CTA-clay sheets.

### 3.2. CTA-Modified Clay/Nanocomposites

#### 3.2.1. XRD Characterization

**Figure 3:**
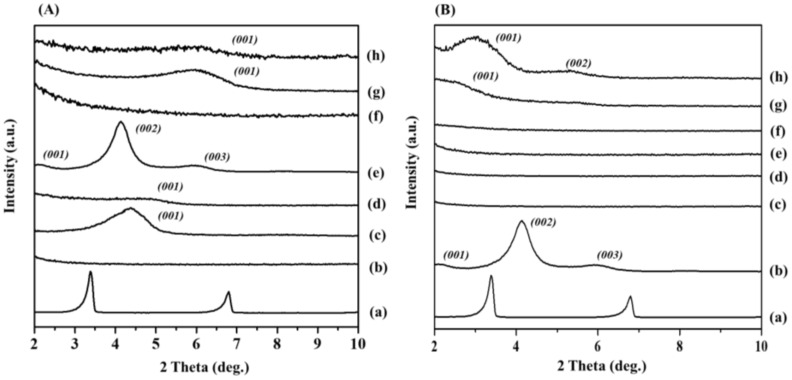
XRD patterns of: (**A**) (**a**) CTA, (**b**) pristine PP, (**c**) CTA-Mt, (**d**) 6 mass % CTA-Mt/PP nanocomposite, (**e**) CTA-Mica, (**f**) 6 mass % CTA-Mica/PP nanocomposite, (**g**) CTA-Ht, and (**h**) 6 mass % CTA-Ht/PP nanocomposite; (**B**) (**a**) CTA, (**b**) CTA-Mica, (**c**) pristine PP, (**d**) 1 mass % (depending on content of CTA-Mica/PP nanocomposite), (**e**) 3 mass %, (**f**) 6 mass %, (**g**) 9 mass %, and (**h**) 12 mass %.

A solution-blending technique was used to prepare the clay-based nanocomposites. For industrial applications, 6 mass % loading of organo-clay is recommended for constructing a polymer nanocomposite. A loading content >6 mass % can lead to low thermomechanical properties because the clays could be agglomerated within the polymer segments. In the previous literature [38,39], it was found that incorporation of >6 mass% of organo-clays lead to poor thermo-mechanical characteristics of the prepared PP nanohybrids. Such experimental findings were observed with 6 mass % mica nanoparticles incorporated in the PP nanocomposites in our studies as well (Figure 3A(f)). It was found that the 6 mass % mica-incorporated PP nanocomposites had well-dispersed mica particles across the polymer chains compared to those with (d) Mt and (h) Ht in nanocomposites. These studies therefore validated our hypothesis that CTA-Mica might be rather completely exfoliated and well distributed uniformly in the PP segments up to 6 mass %. In addition, we attempted to understand how the physical properties of the PP nanocomposites could be changed according to the weight of the nanofiller by comparing and analyzing 1 to 12 mass % by weight of the mica. For optimizing the highest loading value of the organo-Mica for the nanocomposites, they were formed with respect to the various concentrations of the nanofillers (1, 3, 6, 9 and 12 mass %). It was clear that the mica nanofillers were well distributed in the nanocomposites up to 6 mass % (Figure 3B). However, 9 and 12 mass % of mica contents in the nanocomposites were not well dispersed, which was confirmed by the XRD analysis. For example, broad peaks were seen with 9 mass % content (Figure 3B(f), while there were two small peaks seen for the 12 mass % content (Figure 3B(g)), which might have been due to the agglomerated clay particles when their concentration was >9 mass %. In contrast, the broad peaks seen in the XRD pattern of the Mica-PP nanocomposite might have been associated with the self-assembled, CTA-modified mica structures in the nanohybrids. Thus, we concluded that the organo-Mica could be completely exfoliated to have uniform distribution within the PP matrix up to 9 mass % using the solution-blending technique.

#### 3.2.2. TEM Images

**Figure 4:**
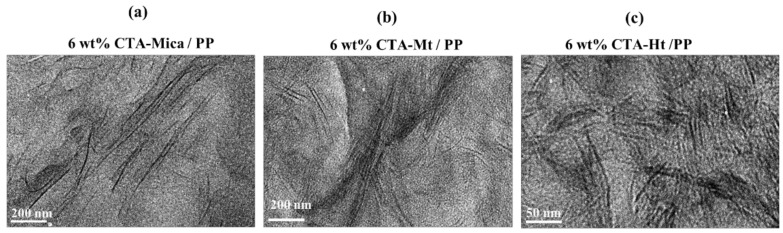
TEM images of (**a**) 6 mass % CTA-Mica/PP, (**b**) 6 mass % CTA-Mt/PP, and (**c**) 6 mass % CTA-Ht/PP nanocomposites.

To further demonstrate the dispersion degree of the various organo-modified nanofillers, a 6 mass % concentration of organo-modified clays/PP composites were analyzed using the cross-sectional TEM images shown in Figure 4. In the case of the 6 mass % CTA-Mica (Figure 4a), the nanosheets were well exfoliated and randomly dispersed without any agglomeration in the PP matrix. An almost similar observation was made for the 6 mass % Mt/PP composites as well (Figure 4b). However, the 6 mass % Ht particles were not fully exfoliated but were dispersed in the PP matrix with irregular stacks of nanosheets (as shown in Figure 4c), which was in good agreement with the XRD results (Figure 3).

#### 3.2.3. Thermal Stability

**Figure 5:**
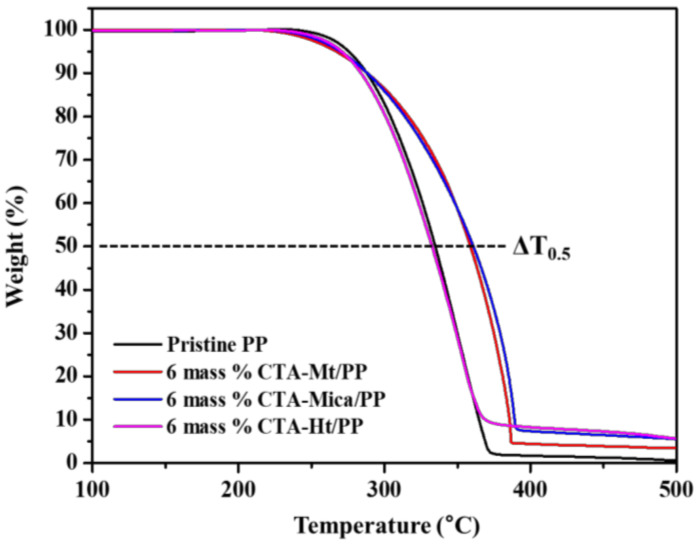
TGA curves for the 6 mass % CTA-clay nanocomposites.

The thermal stabilities of the 6 mass % CTA-clay/PP nanocomposites are represented in Figure 5. The TGA analysis showed that the thermal stabilities of the CTA-Mica and CTA-Mt were increased by including 6 mass % of nanofiller in the PP matrix. Additionally, the thermal decomposition temperature of the PP was substantially increased to a higher temperature after adding 6 mass % CTA-Mt and 6 mass % CTA-Mica. The T_0.5_ (temperature at which 50% of weight loss occurs) of the 6 mass % CTA-Mt/PP nanohybrid was found to be 359 °C, which was 25 °C more than that for the intact PP (334 °C). For mica, the T_0.5_ value was enhanced to 361 °C (ΔT_0.5_ = 27 °C), whereas the T_0.5_ value for the nanocomposite with 6 mass % CTA-Ht was 328 °C with no improvement in the thermostability. Therefore, we chose only the CTA-Mica- and CTA-Mt-containing composites for further studies.

**Figure 6:**
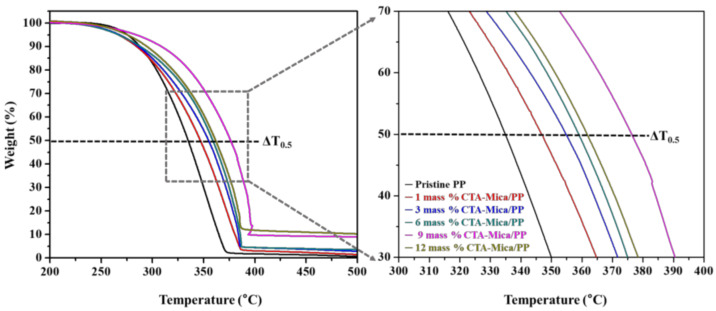
TGA curves for CTA-Mica/PP nanocomposites.

The TGA parameters of T_0.5_ and ΔT_0.5_ (T_0.5_ (nanocomposite)-T_0.5_ (pristine PP)) for the thermal characteristics of the nanocomposites depending on the nanofiller content of each organo-modified mica and Mt (1~12 mass %) are summarized in Table 2. For the organo-Mt modified nanohybrids, the 6 mass % loading resulted in a higher T_0.5_ value, which increased from 334 °C to 359 °C (ΔT_0.5_ = 25 °C). In contrast, the 9 mass % CTA-Mt-containing nanocomposite had its ΔT_0.5_ value reduced by 18 °C, which was related to the XRD results (Figure S3). Due to the agglomeration of CTA-Mt, the dispersibility was lowered. This indicated that there was no thermal stability if it exceeded 6 mass % for CTA-Mt. We found that both the 6 mass % CTA-Mt- and 6 mass % CTA-Mica-containing nanocomposites exhibited a similar thermal stability. However, the 6 mass % CTA-Mica-containing nanocomposite showed a gradual improvement in thermal stability as its concentration increased due to the uniform intercalation of the exfoliated CTA-Mica layers across the PP chains in the nanocomposite (Figure 3B(c–e)). It is worth noting that the 9 mass % CTA-Mica maximized the T_0.5_ value at 376 °C (ΔT_0.5_ = 42 °C), which was very high compared to that found for CTA-Mt (ΔT_0.5_ = 18 °C) (Figure 6 and Figure S4 and Table 2).

The TGA analysis clearly indicated that CTA-Mica-containing nanocomposites (Figure 6) showed a gradual increase in the T_0.5_ by elevating the nanofiller concentration from 1 to 9 mass %, while there was no further enhancement noted after 9 mass %. In addition, the curve corresponding to the 12 mass % CTA-Mica/PP nanocomposite was in between those of 6 mass % and 9 mass %; however, the XRD results (Figure 3B(h)) indicated a rather poor dispersibility for 12 mass %. Therefore, this was evidence that the CTA-Mica could be increased up to 9 mass % when designing a thermally stable nanocomposite for a PP matrix. It is a well-known fact that the thermostability of nano clay/polymers is dependent on the type of clay, surface properties, degree of exfoliation, and intercalatability of the nanofillers. It has already been reported that the thermostability can have direct connection not just to the clay type, but also to their degree of exfoliation and distribution across the polymer segments. Additionally, it should be mentioned that the organo-modification of clays is critical to a higher functionalization with PP through a hydrophobic–hydrophobic chemistry [10,40,41]. Based on this evidence, we were assured that the thermostability of the CTA-Mica-incorporated nanohybrid could be majorly improved by the surface properties and exfoliation within PP chains, along with a high aspect ratio of the nanofillers, as studied previously [42,43].

#### 3.2.4. Mechanical Properties

Figure 6 demonstrates the mechanical characteristics through the elastic modulus and tensile strength for the nanocomposites with varied contents (1 mass % to 12 mass %) of nanofillers (CTA-Mica). Obviously, the mechanical properties were altered by the dispersion degree of the nanofillers in the composites. Accordingly, the Young’s modulus of the nanocomposites were drastically enhanced to 208 ± 6 MPa when the nanofiller content was ~9 mass % compared to the pristine PP (123 ± 2 MPa), while those with 12 mass % were reduced to 164 ± 20 MPa. The improved Young’s modulus, especially with 9% nanofiller content, could surely be due to the uniform molecular distribution of CTA-Mica in the PP chains along with its exfoliated nanofillers with a high aspect ratio [27,28]. In the case of CTA-Mt, the improvement effect was slightly lower than that for CTA-Mica (Figure S5). The Young’s modulus of the CTA-Mt was improved to 160 ± 12 MPa when the nanofiller content was 6 mass % and decreased to 140 ± 11 MPa with 9 mass % when the nanofiller content was 9 mass %. When comparing with the same ratio of 6 mass % CTA clays, the Young’s modulus was 176 ± 12 MPa at 6 mass % for the CTA-Mica/PP, which was higher than that for the Mt (160 ± 12 MPa) and Ht (126 ± 9 MPa). (Figure S6). Such a low mechanical strength for the Mt- and Ht-containing nanocomposites could have been due to their low exfoliation properties compared to those of the mica -clays in the PP segments, as evidenced by the XRD results (Figure 3A(d,f,h)). Of course, the mechanical properties of the nanocomposites could be varied by the type of nanofillers along with several physical properties such as the surface area, aspect ratio, and layer charge (Table S1).

**Figure 7:**
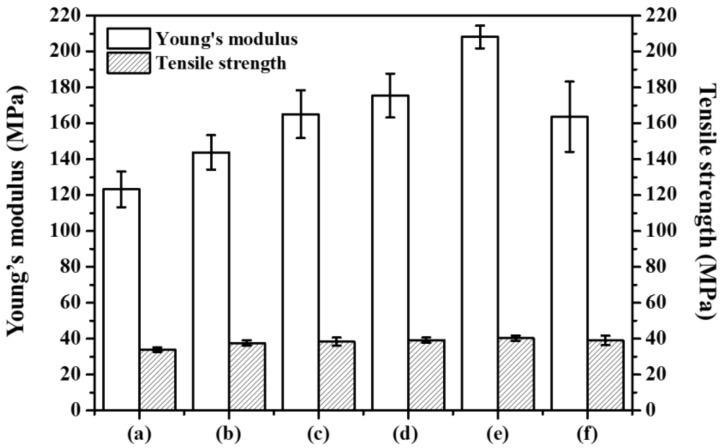
Mechanical properties analysis: Young’s modulus and tensile strength of (**a**) pristine PP, (**b**) 1 mass % (depending on content of CTA-Mica/PP nanocomposite), (**c**) 3 mass %, (**d**) 6 mass %, (**e**) 9 mass %, and (**f**) 12 mass %.

We found that the tensile strength analyses of the CTA-Mica/PP nanohybrids did not make any contribution to the scope of our study (Figure 7). The weak tensile strength might have been due the poor adhesive bonding among the CTA-Mica and PP chains. Similar observations have been already documented by previous researchers as well [10,44,45]. Therefore, we confirmed that that improved mechanical stability of the nanocomposites could be associated with the large surface area of the CTA-Mica nanofillers due to the enhanced van der Waals energy gain between the exfoliated nano-fillers and the host molecules of the PP chains [16,29].

## 4. Conclusions

We were successful in converting hydrophilic smectite clays into organophilic ones via modification with CTA molecules in order to improve the thermomechanical stabilities of PP. It was found that among the three tested smectite clays (mica, Mt, and Ht), the CTA-Mica and CTA-Mt nanofillers drastically enhanced the mechanical properties; whereas the thermal stability was the highest for the CTA-modified mica/PP composites compared to those of both the CTA-Mt and CTA-Ht/PP composites. Such dramatic changes in thermomechanical stabilities of the PP nanocomposites after the incorporation of the CTA-Mica and CTA-Mt nanofillers could be attributed to: (i) layer charges of the CTA-Mica and CTA-Mt; (ii) surface properties and large aspect ratio; (iii) a higher degree of exfoliation and thereby an enhanced homogenous dispersion across the PP chains.

## Figures and Tables

**Table 1:** FT-IR analysis of CTA, unmodified clays, and CTA clays.

Compound	Band Position (cm^−1^)			Assignments
CTA	2930–2840 1470			*ν*(C-H) *ν*(C-N)
	**Mt**	**Mica**	**Ht**	
**Unmodified clays**	3630	3500	3625	ν(Al-OH-Al)
	3416	3000	3421	*ν(H_2_O)*
	1640	1688	1640	*H-bonds*
	1120	1137	1091	*ν(Si-O)*
	1000	1055	956	*δ(Al-OH)*
	796	797	787	*δ(Al-Mg-OH)*
	625	655	651	*δ(Mg-OH)*
	523	526	542	*δ(Si-O-Al)*
	459	436	433	*δ(Si-O, Si-O-Si)*
**CTA Clays**	**CTA-Mt**	**CTA-Mica**	**CTA-Ht**	
	3637	3608	3693	ν(Al-OH-Al)
	3403	3441	3434	*ν(N-H)*
	2930–2840	2935–2840	2927–2853	*ν(C-H)*
	1652	1646	1639	*H-bonds*
	1480	1474	1470	*ν(C-N)*
	1122	1126	1111	*ν(Si-O)*
	1033	994	970	*δ(Al-OH)*
	900–790	707	787	*δ(Al-Mg-OH)*
	624	674	657	*δ(Mg-OH)*
	525	537	531	*δ(Si-O-Al)*
	462	472	479	*δ(Si-O, Si-O-Si)*

ν = Stretching vibrations; δ = bending vibrations.

**Table 2:** Thermal stability of CTA-clay/PP nanocomposites compared to pristine PP.

Sample	CTA Content (mass%)	T_0.5_ (°C) ^a^	ΔT_0.5_ (°C) ^b^
Pristine PP	−	334	−
CTA-Mt/PP	1	333	−1
	3	355	21
	6	359	25
	9	352	18
	12	351	17
CTA-Mica/PP	1	347	13
	3	358	24
	6	361	27
	9	376	42
	12	362	28
CTA-Ht/PP	6	328	−6

^a^ T_0.5_ is the temperature at 50% weight loss; ^b^ ΔT_0.5_ = T_0.5_ (nanocomposite)-T_0.5_ (pristine PP).

## Data Availability

All the necessary information related to this study can be found in the manuscript and in the Supplementary Materials.

## Supplementary Materials

The following supporting information can be downloaded at: https://www.mdpi.com/article/10.3390/polym14194110/s1, Table S1. CEC, particle sizes, layer charges, and aspect ratios of unmodified clays. Figure S1. The molecular structures of (a) cetyltrimethylammonium bromide and (b) polypropylene. Figure S2. The cationic arrangements of (a) CTA-Mt, (b) CTA-Mica, and (c) CTA-Ht. Figure S3. XRD patterns of (a) CTA-Mt (b) pristine PP, (c) 1 mass % (depending on content of CTA-Mt/PP nanocomposite), (d) 3 mass %, (e) 6 mass %, (f) 9 mass %, and (g) 12 mass %. Figure S4. TGA curves for CTA-Mt/PP nanocomposites. Figure S5. Young’s modulus and tensile strength of (a) pristine PP, (b) 1 mass % (depending on content of CTA-Mt/PP nanocomposite), (c) 3 mass %, (d) 6 mass %, (e) 9 mass %, and (f) 12 mass %. Figure S6. Young’s modulus and tensile strength of (a) pristine PP, (b) 6 mass % CTA-Mt/PP, (c) 6 mass % CTA-Mica/PP, and (d) CTA-Ht/PP nanocomposites.
