# Supplementary material for: Effects of Nanofillers Based on Cetyltrimethylammonium-Modified Clays in a Polypropylene Nanocomposite
Source: Polymers (Basel). 2022 Sep 30;14(19):4110. doi: 10.3390/polym14194110 (PMC9573217; doi:10.3390/polym14194110)
Supplement: Supplementary file 1 [file polymers-14-04110-s001.zip › polymers-1918398-supplementary.pdf]

polymers

MDPI

Supporting Information

Effects of Nanofillers based on Cetyltrimethylammonium-Modified Clays in a Polypropylene Nanocomposite

Hyeon-Ju Ryu[1, #], Nguyen Thu Hang[1,#], Sanoj Rejinold. N[2], Byeongmoon Jeong[1], Goeun Choi[2,3.4]and Jin-Ho Choy[2,5,6,7*]**

[1]Department of Chemistry and Nanoscience, Ewha Womans University, Seoul 03760, Republic of Korea
[2] Intelligent Nanohybrid Materials Laboratory (INML), Institute of Tissue Regeneration Engineering (ITREN), Dankook University, Cheonan 31116, Republic of Korea
[3] College of Science and Technology, Dankook University, Cheonan 31116, Republic of Korea
[4] Department of Nanobiomedical Science and BK21 PLUS NBM Global Research Center for Regenerative Medicine, Dankook University, Cheonan 31116, Republic of Korea
[5] Division of Natural Sciences, the National Academy of Sciences, Seoul, Republic of Korea.
[6] Department of Pre-medical Course, College of Medicine, Dankook university, Cheonan 31116, Republic of Korea
[7] International Research Frontier Initiative (IRFI), Institute of Innovative Research,
Tokyo Institute of Technology, Yokohama 226-8503, Japan

# These authors contributed equally to this work;
* Corresponding authors.
Chair Professor Jin-Ho Choy
Tel: +82-41-550-3078
E-mail address: **jhchoy@dankook.ac.kr** (J.-H. Choy).
** Corresponding author.
Professor Goeun Choi
Tel: +82-41-550-3464
E-mail address: **goeun.choi@dankook.ac.kr (**G. Choi)

Table S1. CEC, particle sizes, layer charges and aspect ratios of un-modified clays.

Host	Chemical composition	Diameter of particles (nm)[a]	Cation exchange capacity (CEC) (meq/100g clay)[b]	Layer charge (e^-/unit cell)[b]	Aspect ratio[b]
Mt	$Na_{0.35}K_{0.01}Ca_{0.02}(Si_{3.89}Al_{0.11})(Al_{1.60}Mg_{0.32}Fe_{0.08})O_{10.01}(OH)_2$	~ 1000	115	0.40	250
Mica	$Na_{0.66}Mg_{2.68}(Si_{3.98}Al_{0.02})O_{10.02}F_{1.96}$	~ 1500	120	0.65	1000
Ht	$Na_{0.70}[(Si_{8.00}Mg_{5.50}Li_{0.30})O_{20.00}(OH)_4]$	~ 60	75	0.20	30

[a] The particle sizes measured using a DLS.

[b] Information was obtained from clay industries.

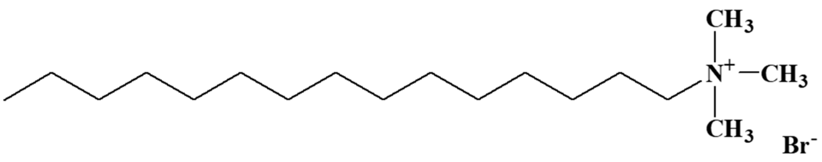

(a) Cetyltrimethylammonium bromide (CTA, $C_{19}H_{42}BrN$)

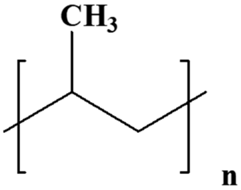

(b) Polypropylene (PP, $(C_3H_6)_n$)

Figure S1. The molecular structures of (a) cetyltrimethylammonium bromide and (b) polypropylene.

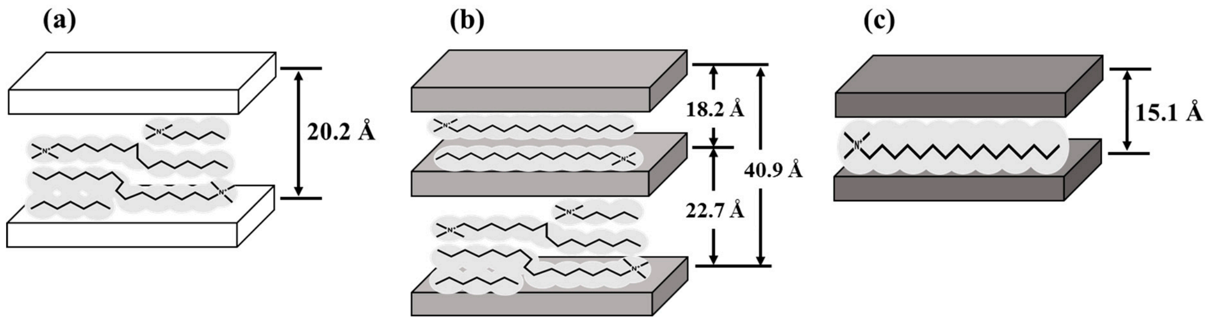

Figure S2. The cationic arrangements of (a) CTA-Mt, (b) CTA-Mica, (c) CTA-Ht.

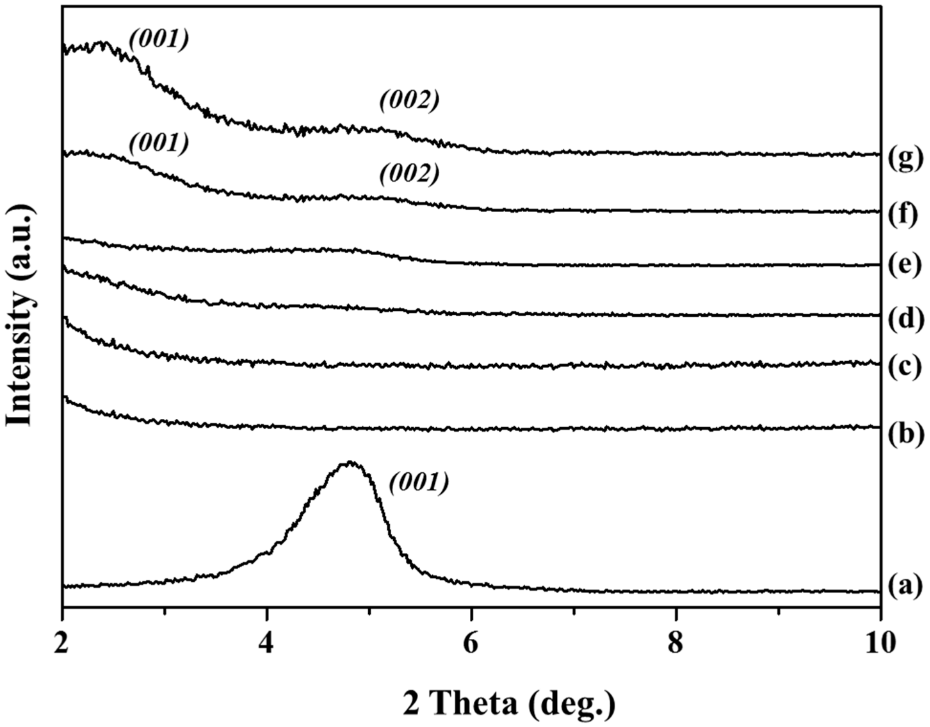

Figure S3. XRD patterns of (a) CTA-Mt (b) pristine PP, (c) 1 mass% (depending on content of CTA-Mt/PP nanocomposite), (d) 3 mass%, (e) 6 mass%, (f) 9 mass%, (g) 12 mass%.

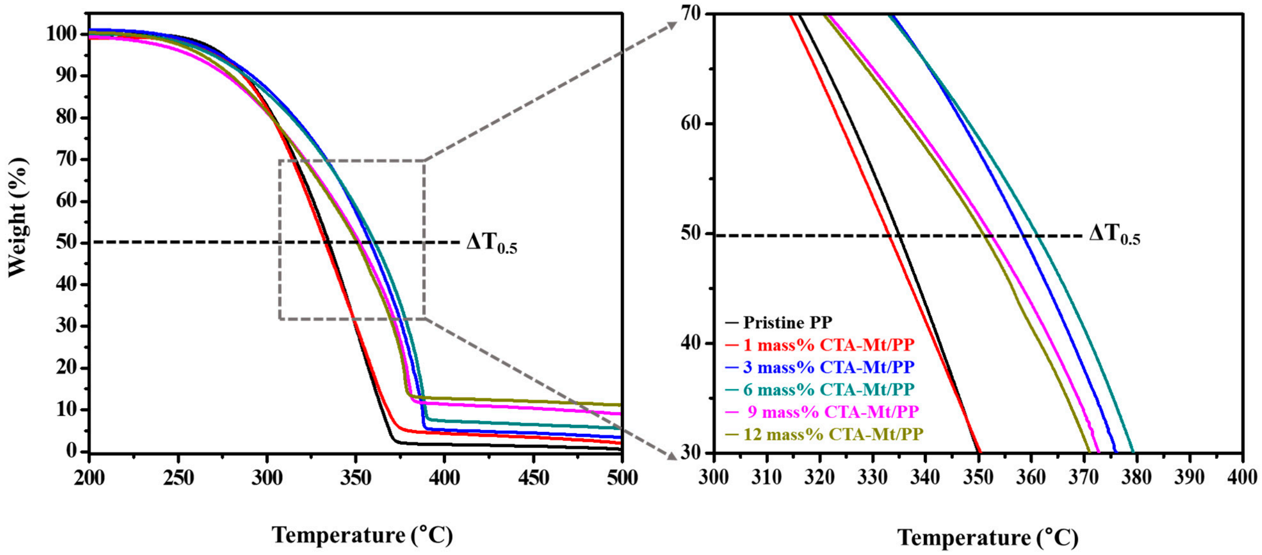

Figure S4. TGA curves of CTA-Mt/PP nanocomposites.

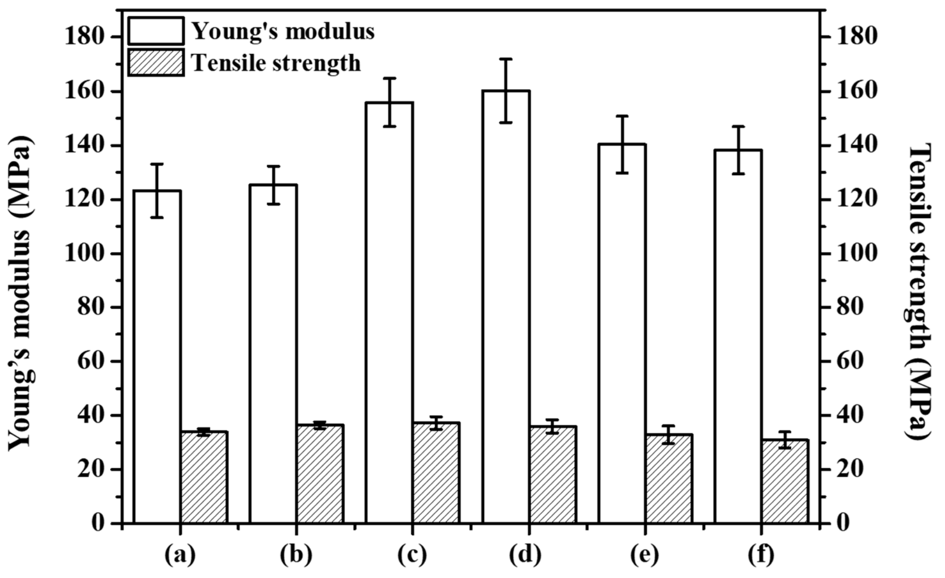

Figure S5. Young's modulus and tensile strength of (a) pristine PP, (b) 1 mass% (depending on content of C TA-Mt/PP nanocomposite), (c) 3 mass%, (d) 6 mass%, (e) 9 mass%, (f) 12 mass%.

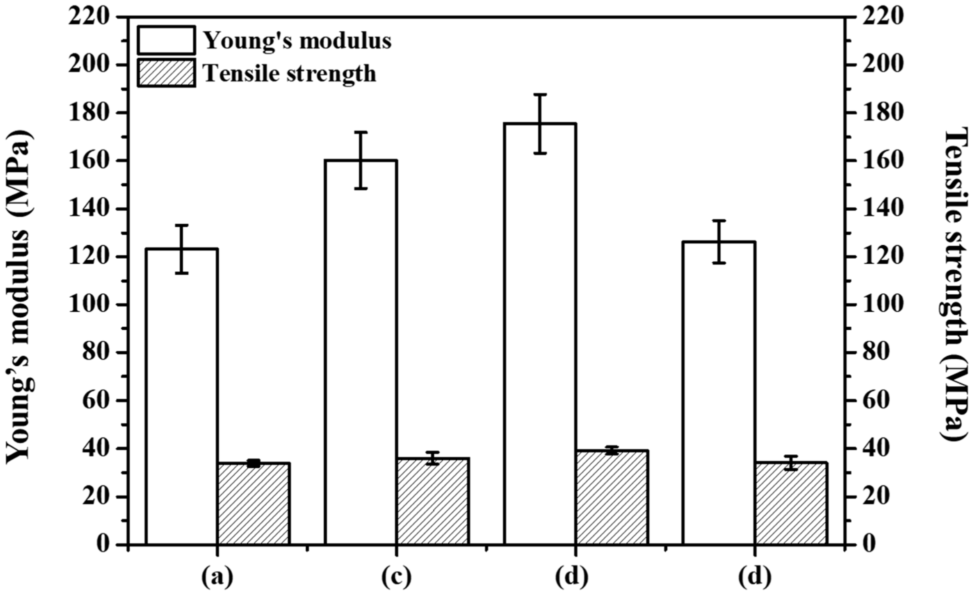

Figure S6. Young's modulus and tensile strength of (a) pristine PP, (b) 6 mass% CTA-Mt/PP, (c) 6 mass% CT A-Mica/PP and (d) CTA-Ht/PP nanocomposites.
